# Analysis of microRNAs and their targets from onion (Allium cepa) using genome survey sequences (GSS) and expressed sequence tags (ESTs)

**DOI:** 10.6026/97320630015907

**Published:** 2019-12-31

**Authors:** Rukmini Mishra, Rupesh Mohapatra, Bijayalaxmi Mahanty, Raj Kumar Joshi

**Affiliations:** 1 School of Applied Sciences, Centurion University of Technology and Management, Odisha, India; 2 Centre for Biotechnology, Siksha O Anusandhan University, Bhubaneswar-751030, Odisha, India; 3 Dept. of Biotechnology, Rama Devi Women's University, Vidya Vihar, Bhubaneswar-751022, Odisha, India

**Keywords:** Allium cepa, microRNA, ESTs, GSSs, miRNA targets, gene ontology

## Abstract

MicroRNAs are small non-coding RNAs of 21-24 nucleotides in length that acts as important modulators of gene expression related to numerous biological processes including development and defense response in eukaryotes. However, only a limited report on onion (Allium cepa) miRNAs is available and their associated role in growth and development of onion is not yet clear. Therefore, it is of interest to identify miRNAs and their targets in Allium cepa using the genome survey sequences (GSSs) and expressed sequence tags (ESTs) and deduce the functions of the target genes using gene ontology (GO) terms. We report 14 potential miRNAs belonging to 13 different families (miR162, miR168, miR172c, miR172e, miR398, miR400, miR414, miR1134, miR1223, miR6219, miR7725, miR8570, miR8703 and miR8752). BLAST analysis using psRNATarget server predicted 39 potential targets for the identified miRNAs majority of which were transcription factors implicated in plant growth, development, hormone signaling and stress responses. These data forms the basis for further analysis and verification towards understanding the miRNA mediated regulatory mechanism in Allium cepa.

## Background

MicroRNAs (miRNAs) are a group of 21-24 nucleotides (nt) small endogenous RNA sequences that acts as negative regulators of gene expression and play significant modulatory roles in numerous biological processes such as growth, development and response to biotic and abiotic stresses [1]. These are basically transcribed out from the endogenous MIRNA genes within the intronic and intergenic regions of the eukaryotic genomes in the form of a stem-loop primary miRNA (Pri-miRNA) structure. The pri-miRNA is processed by Dicer-like 1 (DCL1)/Hyponastic Leaves 1(HYL1)/Serrate Protein (SE) into hair-pin pre-miRNA and subsequently diced out mature miRNA: miRNA* duplex that are exported into the cytoplasm by HASTY1 (HST1) protein. In the cytosol, the mature miRNA from the duplex binds with the endonuclease ARGONAUTE (AGO) protein forming the RNA induced silencing complex (RISC) and accomplish the regulation of gene expression through cleavage or translational inhibition of the target transcript [2]. Although there are different small non-coding RNAs in plants, miRNAs are unique in the sense that, (1) they are specifically encoded by MIRNA genes, (2) possesses a typical stem-loop structure with negative minimal folding free energy (MFE), (3) have a distinct miRNA* sequence, and (4) and exhibit a high degree of sequence complementarity with their specific targets. While a single miRNA can modulate the expression of multiple genes, several miRNAs may also get tangled in the regulation of a specific gene [3]. As such, identification of complementary targets is fundamental to understand the modulatory roles of miRNAs. During the last couple of decades, notableadvancement has been made in exposing the fundamental role of miRNAs in plant growth, hormone signaling, organogenesis, floral differentiation and myraids of stress responses [4].

The mature miRNAs are well-conserved throughthe plant kingdom [5] making it a significant instrument for the identification of novel miRNAs using homology search based comparative genomics approach. Several plant miRNAs have been identified through high throughput computational strategies including direct cloning and next generation deep sequencing.However, in cases where the whole genome sequence is not available, the similarity search using Basic Local Alignment Search Tool (BLASTn) for nucleotide sequences within the highly conserved regions of the pre-miRNAs and mature miRNAs as well as matching of the secondary hairpin structure could be effective criterion for miRNA identification in plant species. This is possible by making use of multiple data source including the expressed sequence tags (ESTs) and genome survey sequences (GSSs). Computational analysis of ESTs and GSSs predicted miRNAs with greaterprecisionin various plant species including Arabidopsis [6], wheat [7], maize [8], sorghum [9] and finger millet [10].The latest release of miRBase (release 22) database has reported 7385 mature miRNA sequences within the Viridiplantae miRNA dataset including 713 from O. sativa, 756 from Medicagotruncatula, 401 from Populustrichocarpa, 321 from Zea mays and 241 from Sorghum bicolor.

Bulb onion (Allium cepa L.) is an economically important vegetable crop cultivated in greater parts of the world. Besides being an ingredient with high food value, onion is also credited with numerous medicinal properties including for the treatment of cardio-vascular disorders, chicken pox, measles and myraids of cancers [11]. As per global data, onion is one among the five most important fresh market vegetable crops [12]. India is the second largest producer of onion with an area of 0.52 million hectare producing about 6.50 million tonnes. However, the productivity of onion is gradually decreasing complemented with price rise due to several environmental factors such as drought, salinity and biotic stresses including infection by pests and pathogens [13]. Emerging evidences specify that miRNAs and the related RNA interference pathway components are significant elements in the modulation of plants response to biotic and abiotic stresses [4]. A systematic study of miRNAs and their targets in onions could provide novel perceptions into the molecular and biochemical mechanisms of onion development, growth and response to environmental stimuli. Onion contains ESTs and GSSs deposited in the National Centre for Biotechnology Information (NCBI), which could be used as the starting material for predicting miRNAs in this economically important plant species. A previous study had reported 9 onion miRNAs using the ESTs datasets [14]. In the present study, we used a robust homology based comparative algorithm approach for the detection of onion miRNAs and their targets from the EST and GSS datasets. Further, the target genes of the identified miRNAs were also functionally annotated to understand their role in plant development and metabolic processes.

## Methodology

### Sequence database and reference set for miRNA identification:

All mature miRNA sequences from Viridiplantae group were reclaimed from the miRNA database miRBase (http://www.mirbase.org/) [15]. All these mature miRNAs were previously resulted from different plant species by initial computational identification followed by validation through different experimental approaches including cloning, sRNA sequencing, northern blotting and qPCR method. Mature miRNAs were made non-redundant by duplication to prevent overlapping of miRNA sequences. Taking all these unique mature miRNA sequences as reference, our target miRNA sequences were identified from onion ESTs and GSSs by homology search method. Publicly available 20204ESTs and 10725 GSSs (as of December, 2019) of onionwere downloaded from (NCBI) (www.ncbi.nlm.nih.gov/) by using keyword "Allium cepa".

### Prediction of A. cepa miRNA:

**Figure 1:**
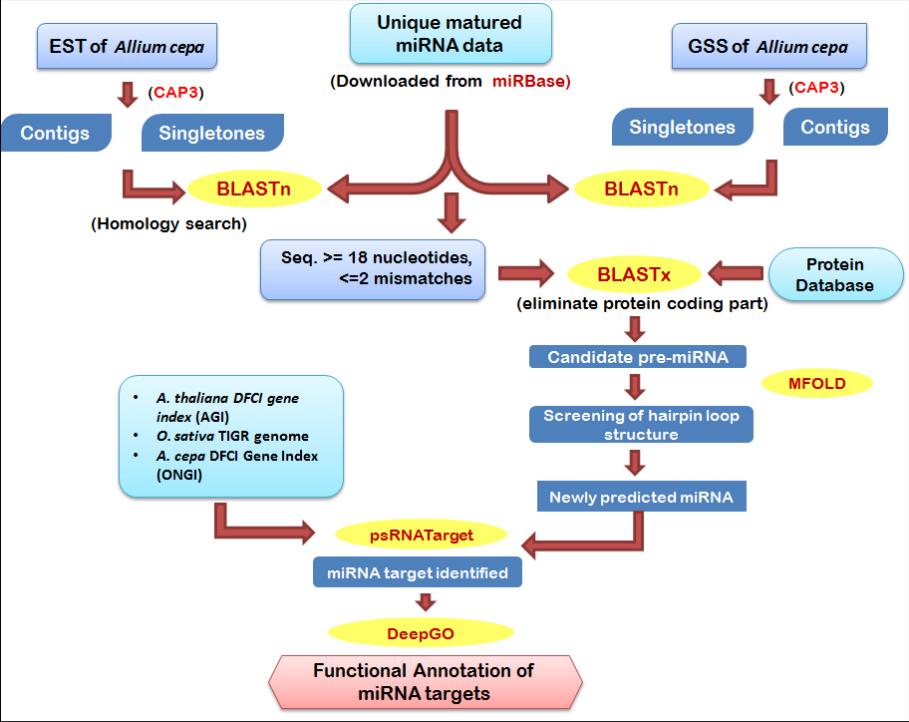
Schematic approach for the prediction of miRNAs and their targets from ESTs and GSSs of Allium cepa.

Prediction process of putative miRNA fromAllium cepa is represented in (Figure 1). Sequences from the locally developed onion EST and GST databases were BLAST searched against the GenBank database (www.ncbi.nlm.nih.gov/genbank) and Rfam database ver 12.0 (www.rfam.xfam.org). The resulted sequences were further analyzed withBLASTx [16] to identify and eliminate the coding sequences.The filtered sequences were used for homology search against the known mature and non-redundant plant miRNAs in miRBase (Release 22; http://www.mirbase.org/search.shtml).Sequence alignment of the ESTs and GSSs against the known miRNAs was retrieved throughBLASTn algorithmswith a threshold E value of 10, sequence filtration at low complexity and word match size between the query and the database set at 7.Homologous candidate miRNAs were identified based on following parameters: EST/GSS sequences with a miRNA matching region of 18 nucleotides with no gap, and base mismatch between predicted sequences and the known miRNAs should be ≥ 3.Zuker algorithm in the MFOLD program predicted the secondary loop structures of the miRNA precursors [17].The hairpin structures of the precursors were confirmed using the following criteria: hairpin should have atleast 18nt mature miRNA in one arm of the stem loop; 50% of bases should be paired; <4 nt bulge between miRNA and miRNA*; minimum bulge size of 1 or 2 bases and 1 or less asymmetric bulges within the miRNA/miRNA*;30-70% contents of A + U and high negative MFE and minimal foldingfree energy index (MFEI) of predicted secondary structure.Negative MFE value of each potential precursor miRNAs were determined by theΔG values (-kcal/mol) of stem-loop structures, which is directly correlated with the sequence length [5].MFE of a 100 nucleotide length is represented as adjusted minimal folding free energy (AMFE) and is calculated as: AMFE = [MFE / length of precursor sequence (LP)] x 100. Subsequently, the MFEI was calculated as MFEI = AMFE /(G + C) % [5].

### Prediction of potential miRNA targets:

Presumed targets for candidate onion miRNAs were predicted using the psRNA-Target webserver (http://plantgrn.noble.org/ psRNATarget/) [18]. Pairwise sequence similarity analyses was performed by querying the A. thaliana DFCI gene index (AGI) release 15, O. sativaTIGR genome cDNA OSA1 and A. cepa DFCI Gene Index (ONGI) cDNA library sequences with the mature onion miRNAs. The default parameters for prediction of miRNA targets are: miRNA-target mismatches ≤ 2; complementary scoring length of 20; maximum energy allowed to unpair the target is 23; flanking length around the target for accessibility (17bp upstream/13bp downstream); range of central mismatch for translational inhibition was set at 9-10nt; number of target for each miRNAs was set at 10.

### Phylogenetic analysis of onion miRNAs:

The predicted mature onion miRNAs were subjected to nucleotide research using BLASTnagainst all plant miRNAs as query with a default setting of 3bp mismatch and threshold E value of 0.001. The homologous miRNA precursor sequences were identified and retrieved from miRBase. Multiple sequence alignment of the identified miRNA precursor sequences along with the collected precursors from other plant species wasperformed using Clustal Omega (https://www.ebi.ac.uk/Tools/ msa/clustalo/) with default parameter and manually adjusted using BioEdit 7.1 software. Phylogenetic analyses of the miRNA sequences were performed using Molecular Evolutionary Genetic Analysis (MEGA v 10.1) package [19].A neighbour joining (NJ) method with 1000 bootstrapping was performed to develop an uprooted phylogenetic tree.

### Functional annotation of the miRNA targets:

The functional aspects of the miRNA targets are crucial to comprehend the range of miRNA regulation in the biochemical and molecular mechanism of plant growth and development.Functional enrichment of the miRNA targets was performed using the Blast2GO v3.0 [20] and further verified using the DeepGO prediction tool with the protein GO classes [21].Identified target genes were categorized in terms of molecular functions, biological processes and cellular components.

## Results and Discussion:

Majority of miRNAs are evolutionarily conserved throughoutthe plant kingdom and therefore could bereadily exploited for detection of miRNAs from the ESTs and GSSs of plant species whose complete genome sequence is not yet available [5]. A homology based comparative genome approach was used in the present study for identification of miRNAs from A. cepa, a plant with great culinary and economic significance.A total of 7385 non-redundant eukaryotic miRNA sequences from the clade of Viridiplantae were used as reference query for BLAST search against 20225 ESTs and 10725 GSS sequences of A.cepa. BLASTn analysis of 503 assembled contigs and 27190 singletons resulted in 54 non-redundant sequences exhibiting high degree of similarity with the Viridiplantae miRNA reference set. BLASTx search against the NCBI protein database identified 29 sequences with protein coding potential and were subsequently eliminated. The remaining 25 sequences were assessed for their ability to develop secondary structure using MFOLD software. A strict evaluation of the hairpin structure predicted only 14 potential miRNAs (6 from ESTs and 8 from GSSs) demonstrating significant sequence identity with conserved Viridiplantae miRNA (Table 1). A recent study carried out by Kohnehrouz et al. [14] reported the identification of 9 miRNAs and their targets from A. cepa ESTs. In contrast, we identified 14 miRNAs from A. cepa ESTs and GSSs and predicted their detailed functional properties. Interestingly, all except one miRNAs (ace-miR414) were new and completely different from those that were identified earlier. This may be attributed to the fact that, both the EST database and the GSS database of A. cepa was used for miRNA prediction in the present study. Of the 14 miRNAs, 6 were predicted from EST sequences (2.96% per 10,000 ESTs) and 8 were identified from GSSs (7.45% per 10,000 GSSs).These values are quite significant as compared to miRNA candidates from other plant species [5] and confirmed that EST and GSS analysis could be efficiently used to predict miRNAs in plants. Among the newly identified potential A. cepa miRNAs, only ace-miR172 consisted of two members (ace-miR172c and ace-miR172e) while the remaining 12 of them (ace-miR162, ace-miR168, ace-miR398, ace-miR400, ace-miR414, ace-miR1134, ace-miR1223, ace-miR6219, ace-miR7722, ace-miR8570, ace-miR8703 and ace-miR8752) were represented by single member only.miR1223, miR6219, miR7722, miR8570, miR8703 and miR8752 from A.cepa were uniquely reported earlier in Physcomitrella patens [22], Sorghum bicolour [9], Brachipodium distachyon [23], Amborella trichopoda [24] and Gossypium raimondii [25]. Kohnehrouz et al.[14] identified three unique miRNAs-miR1440, miR2673 and miR5021 from A.cepa ESTs. The presence of several unique miRNAs in A.cepa suggests that they could be involved in the modulation of specific functions related to growth, development and stress responses.

**Figure 2:**
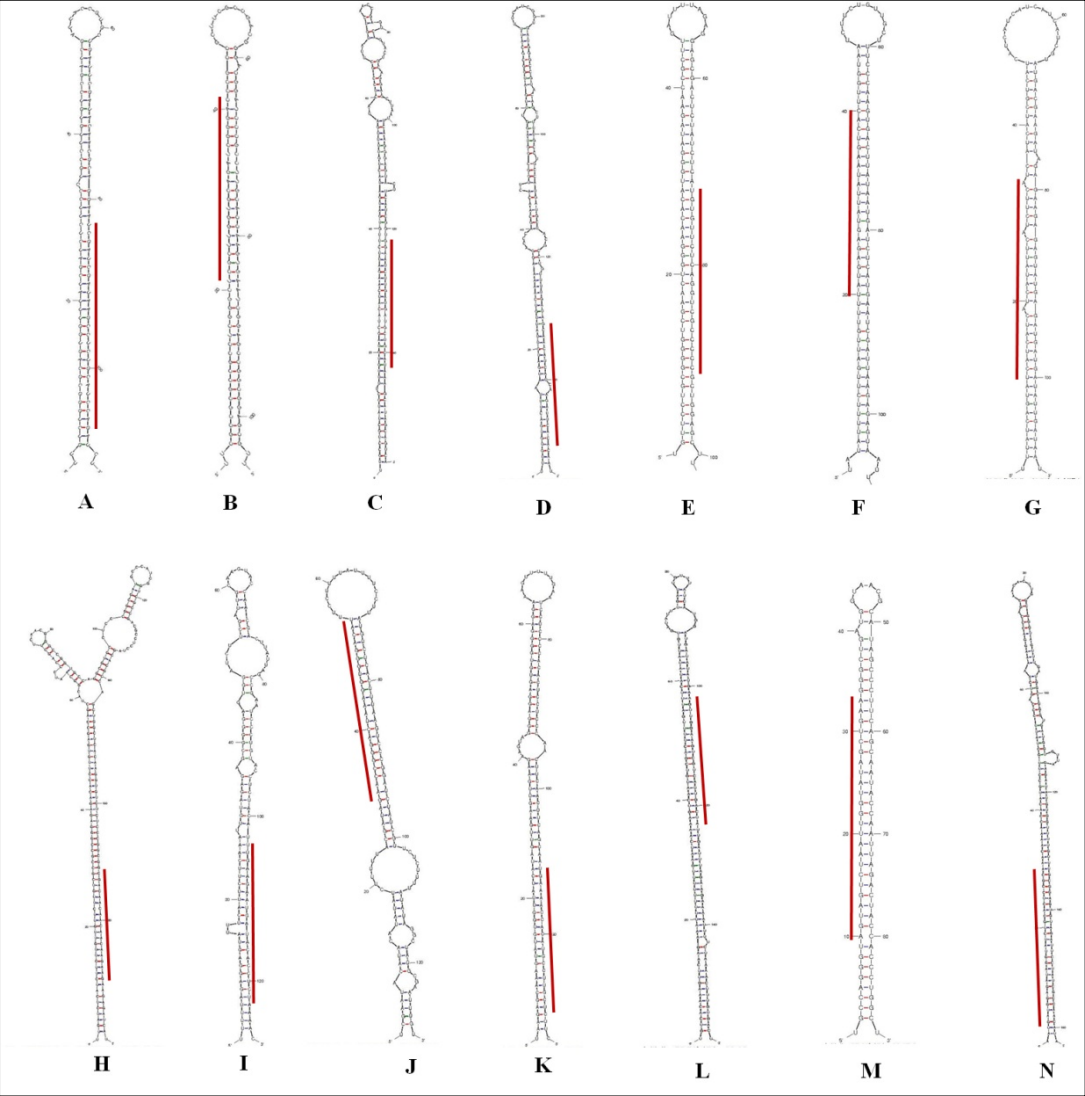
Representative hairpin secondary structures of the predicted onion miRNAs as generated by the MFOLD program. Mature miRNAs are indicated with red lines. (a) ace-miR162, (b) ace-miR168, (c) ace-miR172c, (d) ace-miR172e, (e) ace-miR398, (f) ace-miR400, (g) ace-miR414, (h) ace-miR1134, (i) ace-miR1223, (j) ace-miR6219, (k) ace-miR7725, (l) ace-miR8570, (m) ace-miR8703 and (n) ace-miR8752.

Sequence analysis of miRNA precursors showed that the length of A.cepa pre-miRNA and mature miRNA varied from 89 to 200 and 21 to 24, respectively. Plant miRNA biogenesis have shown that the length of plant pre-miRNAs ranges between 100 nt to 1000nt and are usually longer than the animal pre-miRNAs [26].Our result corroborated with the previouslydescribed miRNAs and their precursors in Arabidopsis [27], rice [28], maize [8] so also in A. cepa [14]. The identified A. cepa miRNAs and their flanking sequences exhibited the typical hairpin loop structures (Figure 2).Earlier reports have shown that, miRNA precursors have lower folding free energyto maintain the thermodynamic stability of the hair-pin loop [29].The newly identified onion miRNA precursors exhibited negative MFE values ranging between -48.71 to -142.30 with an average of -76.79±26.81 Kcal/mole (Table 1 and Table 3). As the length of the miRNA precursor is directly related to MFE value, often the average folding energy (AMFE) and the minimal folding free energy index (MFEI) were calculated to standardise the potential effect of sequence length and differentiate miRNAs from other non-coding RNAs [5].Usually, the MFEI of the miRNA is significantly higher (>0.85) than other RNAs including rRNA (0.59), tRNAs (0.64) and mRNAs (0.62-0.66) [5]. The AMFE of the predicted onion miRNAs ranged from -36.31 to -75.28 with an average of -52.8±15.17 Kcal/mol. While the previously identified onion miRNAs had average MFEI of 0.67±0.15, the miRNAs identified in the present study had MFEI between 0.89 to 1.8 (1.25±0.31) clearly suggesting that these are precisely distinct and true miRNAs.

Analysis of the nucleotide composition in the pre-miRNAs revealed that uridine (U) is the most representative nucleotide with an average of 30.46% followed by adenine (26.75%), guanine (22.18%) and cytosine (20.57%) (Table 2 and Table 3).Twelve out of fourteen identified pre-miRNAs consisted of more than 25% of U as has been reported in the miRNAs of other plant species [8],[30]. It was also observed that 8 out of 14 mature miRNAs had U as the preferred 5' end nucleotide concurring with precedingstudiescarried out in canola [31], garlic [32] and chilli [30]. Also, the (G+C) % and the (A+U) % ranged from 26.41-60.5% and 39.5-73.5% respectively. The (A+U) % is well within the range of 30-70%, which is the gold standard for identification of potential plant miRNAs [26]. All the mature miRNAs were located within the stem of the hairpin loop (Figure 2). While 5 miRNAs (41.6%) were positioned in the 5' end of the secondary structure, the remaining 9 (64.28%) were found in the 3’ end. This corroborate with the previous finding that majority of onion miRNAs are located on the 3' arm of the secondary hairpin loop structure [14].

**Figure 3:**
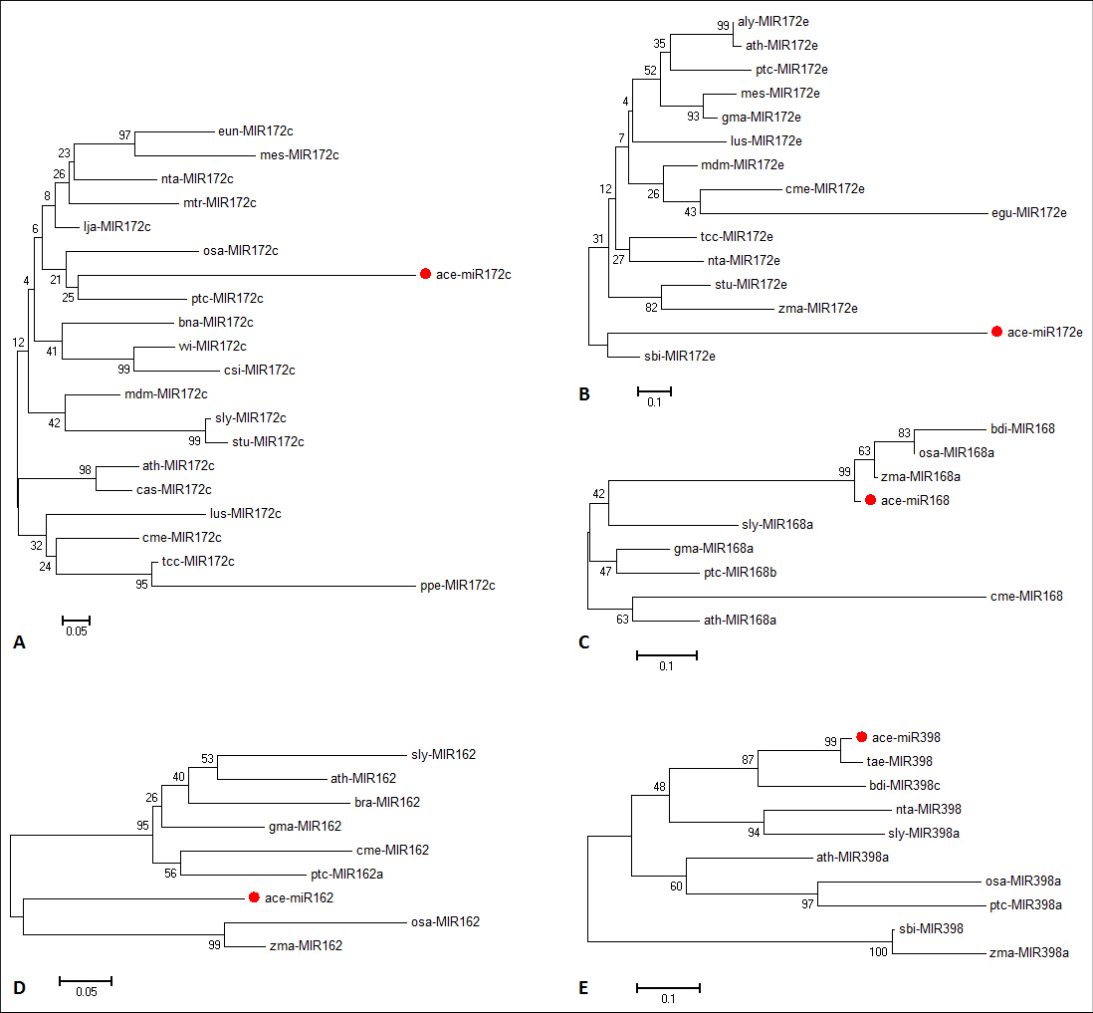
Neighbor-joining phylogenetic tree of the predicted pre-miRNA sequences with their closely related miRNA families from other plant samples. (A) ace-miR172c, (B) ace-miR172e, (C) ace-miR168, (D) ace-miR162 and (E) ace-miR398

Unrooted neighbor-joining phylogenetic tree was developed from the multiple sequence alignment of the identified onion miRNAs and other members of the same family available in miRBase to determine the evolutionary relationship among them. Distinct phylogenetic trees were obtained for ace-miR162, ace-miR168, ace-miR172c, ace-miR172e and ace-miR398 exhibiting high degree of sequence similarity with miRNAs from other plant species (Figure 3). ace-miR172c demonstrated 27-54% of sequence similarity to other previously reported miRNAs having maximum closeness with osa-miR172c of Oryzasativa and ppe-miR172c of Prunuspersica. On the other hand, ace-miR172e precursor was highly similar to sbi-miR172e from Sorghum bicolor followed by zma-miR172e from Zea mays. The percentage similarity between ace-miR162 precursor and miR162 from other plant species ranges between 20-79%. The highest similarity of ace-miR162 precursor was found with osa-miR162 and zma-miR162. Similarly, ace-miR168 precursor demonstrated 55.4% similarity with bdi-miR168 from Brachypodium disthachion followed by 40.4% similarity with zma-miR168 and 36.4% likeliness with osa-miR168. Phylogenetic analysis of ace-miR398 showed that tae-miR398 from Triticumaestivum and bdi-miR398c have significant evolutionary linkage with ace-miR398. Interestingly, ace-miR162, ace-miR168 and ace-miR398 exhibited greater likeliness with members of the same family in monocots. However, no such specific conditions were observed in case of ace-miR172c or ace-miR172e. This suggests that the evolutionary relationship of ace-miRNA is significantly different and is more inclined towards monocotyledonous plants.

miRNA modulate the expression of target mRNA through complementary binding and consequent cleavage and/or translational inhibition [33]. To understand the functional role of the identified onion miRNAs, potential miRNA target genes were predicted using the psRNATarget webserver with default parameters. As the target sites of plant miRNAs are mostly situated in the open reading frames (ORFs) of the target genes, onion ESTs in addition to AGI and TAIR databases were used to search for putative target genes. Based on acceptedprinciples, the algorithm predicted 39 potential target for 13 miRNAs (Table 4). Unsurprisingly, most of the target were similar to the one that have been earlier validated as plant miRNA targets in other plant species including Arabidopsis, rice, wheat, maize and garlic [32]. Nine miRNAs (ace-miR162, ace-miR168, ace-miR172c, ace-miR172e, ace-miR398, ace-miR400, ace-miR414, ace-miR1134 and ace-miR6219) were predicted to have targets in the range of 2 to 8 suggesting that these miRNAs might have diverse functional attributes. Among the 39 targets, 10 were transcription factor (TF) genes including ethylene response factors (ERFs), MADS-Box TFs and Ring-H2 proteins that have been previously implicated in plant growth regulation and development [34]. Most of the targets exhibited high homology with targets from other plants and presumably demonstrated functional redundancy across plant species. For example, ace-miR162 targeting Dicer-like (DCL) proteins, ace-miR168 targeting Argonaute 1 (AGO1) proteins, ace-miR400 targeting pentatrico peptide repeat protein 1 (PPR1) and ace-miR1134 targeting receptor protein kinase PERK1 have been formerly involved in gene regulation and small RNA biogenesis, plant growth, stress response, hormone signalling and host-microbe interactions [31],[32],[35]. ace-miR172c targeted two genes encoding floral homeotic protein APETALA2 suggesting its involvement in the speciation of onion flowers. A few targets were non-transcription factors such as phosphoenol pyruvate carboxylase (ace-miR1134), protein phosphatase (ace-miR8752) and ribosome inactivating protein 1 (ace-miR6219) vindicating their functional role related to plant metabolism, immunity and defense response [36]. Additionally, 8 genes targeted by ace-miRNAs were uncharacterized with unknown function, suggesting that they could be part of unknown biochemical and molecular mechanisms essential for growth and survival of the plant.

To delineate the comprehensive network of genes modulated by miRNAs, the identified targets were subjected to gene ontology (GO) term analysis using the Blast2Go program. A total of 31 out of 39 predicted targets were categorized into 8 biological processes, 5 molecular functions and 4 cellular components (Table 5). Among the biological processes, genes involved in metabolic process (10), secondary metabolic process (5), signaling (5), regulation of transcription (5) and response to stress (4) were mostly represented. Two target genes (CF451171, CF452115) were specifically involved in immune system process (GO: 0002376) and defense response (GO: 0006952).Likewise, transcription factor activity (GO: 0003700; 7 genes), Catalytic activity (GO: 0003824; 7 genes) and nucleic acid binding (GO: 0003676; 6 genes) were the most represented GO terms in the molecular function category. As regards to the putative target transcript of miRNAs in the cellular component category, the GO term cell part (GO: 0044464) was most represented with 5 genes followed by intracellular part (GO: 0044424) and organelle (GO: 0043226) with 3 genes each. The diversified function of these target genes suggest that the complementing miRNAs presumably plays important modulatory role in the signalling, growth, development and defense response to myraids of stresses in onions.

## Conclusion

In conclusion, a comprehensive computational analyses of onion ESTs and GSSs were performed in the present study to identify 14 potential miRNAs belonging to 13 different families. Phylogenetic analysis of the identified miRNAs confirmed their close homology with conserved miRNAs from other plant species. A total of 39 potential targets were predicted for the identified miRNAs with an inhibitive expressional response due to miRNA mediated cleavage or translational repression. Bulk of the predicted target genes encoded transcription and regulatory factors that are implicated in plant growth, development, hormone signalling and stress responses. GO annotation of the target genes revealed that the miRNAs and their associated components are significant modulators of metabolic processes, plant immunity and defense response. These datawill form the basis for further characterization of miRNAs through transient over-expression and knockout study towards exploration of miRNA mediated regulatory mechanism in onion.

## Figures and Tables

**Table 1:** List of predicted miRNAs from Allium cepa and their secondary structure analysis

miR-name	EST/GSS ID	Length of EST/GSS	Length of precursor miRNA	Length of mature miRNA	Position	Strand	No. of mismatches	% GC	MFE	AMFE	MFEI
ace-miR162	ET648103	892	111	21	3'	+	0	50.45	-56.6	-50.99	-1.01
ace-miR168	ET648234	930	105	21	5'	+	0	56.92	-60.5	-57.61	-1.01
ace-miR172c	CF452212	838	158	21	3'	+	0	41.77	-67.1	-42.47	-1.02
ace-miR172e	CF452213	766	155	21	3'	+	0	43.87	-60.2	-38.84	-0.89
ace-miR398	ET647923	823	101	21	3'	+	0	54.45	-65.7	-65.04	-1.19
ace-miR400	ET641297	694	107	21	5'	+	0	38.97	-49.1	-45.88	-1.17
ace-miR414	CF440830	614	111	21	5'	+	0	31.81	-48.71	-43.88	-1.38
ace-miR1134	CF439915	428	200	24	3'	+	0	60.5	-142.3	-71.15	-1.18
ace-miR1223	ET639603	848	127	21	3'	+	3	39.37	-51.3	-40.39	-1.03
ace-miR6219	ET648351	1056	130	24	5'	+	0	35.38	-47.2	-36.31	-1.03
ace-miR7725	CF452205	679	134	21	3'	+	3	44.02	-91.6	-68.36	-1.55
ace-miR8570	ET640090	831	159	22	3'	+	2	26.41	-75.7	-47.61	-1.8
ace-miR8703	ET643849	857	89	24	5'	+	0	48.31	-67	-75.28	-1.56
ace-miR8752	CF452205	679	162	21	5'	+	0	46.29	-88.7	-54.75	-1.18

**Table 2:** Homologs of onion miRNAs and structures of pre-miRNA sequences

miRNA	Homolog miRNA	Mature miRNA Sequence	Homolog miRNA sequence	Precursor miRNA sequence	A%	U%	G%	C%	A/U	G/C
ace-miR162	osa-miR162	UCGAUAAGCCUCUGCAUCCAG	UCGAUAAGCCUCUGCAUCCAG	UUGUCUGGGCGCAGUGGUUUAUCGAUCUCUUCCCUGCCUUGUGUCCGAUCGAUUACCGUGCUGAUUCGAUACAACGCAGGAAUCGAUCGAUAAGCCUCUGCAUCCAGAUCU	18.9	29.7	23.42	27	0.636	0.867
ace-miR168	zma-miR168a	UCGCUUGGUGCAGAUCGGGAC	UCGCUUGGUGCAGAUCGGGAC	UUCGCCGCGCCGCCUCGGGCUCGCUUGGUGCAGAUCGGGACCCGCCGCUUCGCCGACGGGACGGAUCCCGCCUUGCACCAAGUGAAUCGGAGCCGGCGGAGCGUU	12.3	16.19	35.23	36.19	0.759	0.973
ace-miR172c	ata-miR172c-3p	GGAAUCUUGAUGAUGCUGCAU	GGAAUCUUGAUGAUGCUGCAU	UUGCCUGCUAUGCUUUUGUGUAGCUACAUCAAGAUUCUUGUGAUGAUACUGUUUUGAUACUCCGCUUAUGUCAACCGAGUCAGUACUGCAGGAGCUUAAUUCAAGGGAGUACUUCAUCGGUGGAAUCUUGAUGAUGCUGCAUAAAGCAUAGCAGUCAU	24.68	33.54	22.78	18.98	0.735	1.2
ace-miR172e	stu-miR172e-3p	GCAACAUCAUCAAGAUUCACA	GCAACAUCAUCAAGAUUCACA	UUUGUGUGACUUUAAUGAUGUUAUUGUCAAAAAGGACCCAGCA	24.51	31.61	23.22	20.64	0.775	1.125
				ACACUUGGCGGUAGGUUUGAUGGCGCAUUUUCAUCGGUGAUGGGCCGUACAGCUUUUGCCUGCCAUGAUGCUACGCCUGUUUUGAUAGCAACAUCAUCAAGAUUCACACAAU						
ace-miR398	tae-miR398	UGUGUUCUCAGGUCGCCCCCG	UGUGUUCUCAGGUCGCCCCCG	UGUUCCUGCGGGGUCGAACUGGGAACACAUGGGAUAGAACCGCUUGAUUUUAGAGGAGCGACUCUAUCUCAUGUGUUCUCAGGUCGCCCCCGCUGGAGCUU	18.81	26.73	29.7	24.75	0.703	1.2
ace-miR400	bra-miR400	UAUGAGAGUAUUAUAAGUCAC	UAUGAGAGUAUUAUAAGUCAC	UAUAUUUUCUUGAUUGUUUUAUGAGAGUAUUAUAAGUCACUUGGUAAUCUUCUGUUGCUUUCCCAAGUGACUUAUAAUGAUCUCAUGAAUCGAUUAAGAGAGUAAUU	28.03	42.99	16.82	12.14	0.652	1.38
ace-miR414	ath-miR414	UCAUCAUCAUCAUCAUAUUCA	UCAUCAUCAUCAUCAUCUUCA	UUUAUCAGUAUCAUCAUCAUCAUAUUCAUCUUCAUCAUCAUCGUCAUCAUCAUCAUCAUCAUCGUAUGACGAAGAUAGAGAAGAGAGUAUGAGAUGAUGAUAUUGAUAAU	34.23	33.3	14.41	17.11	1.02	0.842
ace-miR1134	far-miR1134	CGACAACAACAACAAGAAGAAGAG	CGACAACAACAACAAGAAGAAGAG	UUGAUCUCAUCUUCUUGUUGUUCUUGUCGCCGCCGGGAGAGACACCUUGUGAGCCAGCUACGGCAAUCCCAGCUUCAACGGGCCGGUGCCAGGCGUGCCUCCCGGCGAUGCCCAUCGGUCGCCGACGCCUCCUAGCACGCCAGCUGGCUCACAAGGUGUCUCUCCCGGCGGCGACAACAACAACAAGAAGAAGAGAUCAU	21	19.5	26.5	33	1.076	0.803
ace-miR1223	ppt-miR1223	UUGAAGAAUGAUACACCUCUA	UUGUAGAGUCAUACACCUCCA	UUUUUAGAGGAGUAUUUCAUUCUUCAAAUUGUAGAGAGCGGGGAGUGGAUCUUCGAUUGCAAGUACCAACGACUUAUUAGCCGAAUCCCUGUACCUCUACAUUUGAAGAAUGAUACACCUCUAAAAC	29.92	30.7	19.68	19.68	0.974	1
ace-miR6219	sbi-miR6219-5p	GAACCGGGACUAAAGGUGGGACAU	GAACCGGGACUAAAGGUGGGACAU	UCGAAUACAUACAUAUAUCUUUUUACGAUAGAACCGGGACUAAAGGUGGGACAUUUCCUUUUAUUUUCCUUAUGUCUCACUUUAAGUCCCGGAUCUAUCGUUCUCUUUUAUAUAGGCUAUGCGAAUUUGU	24.61	40	16.15	19.23	0.615	0.84
ace-miR7725	bdi-miR7725b-3p.1	UGAAAACCACAUGCCUUGCUC	UGAAAACCAUAUUCCUAGCUC	UAAGAGCAAAGUAUGUGGUUAUCAUAGUUGAAGUUGAAGAAGUAGGGAGGAGUGUGGGGAGGAAAGUUUUUGAAUCCUCCCCACACUCCUCCCAACUUCAACUUCAGCAAUGAAAACCACAUGCCUUGCUCUUC	29.85	26.11	23.13	20.89	1.142	1.107
ace-miR8570	aur-miR8570	AGUUGGAAGUCGUCAUUGACUA	AGUUGGGAGUGCGUCAUUGACUA	UGUCACAAAGUUCAAGAAAUUUUUUUUUGAAUCAAAAGUCAAUGUCGACUUUUGAUUUCAUAUAUUGUGAUAUUUGAUUGUUUAUCAUGGAUGAUAUAUGAAGUUGGAAGUCGUCAUUGACUAUUGAUUUAGAAGAAAAUUUUCUAAAACUUUGUGACU	32.07	41.5	16.98	9.43	0.772	1.8
ace-miR8703	gra-miR8703b	AGUAGUCUAAUUGGUAUAGCUGAA	AGUAGUCUAAUUGGUAUAGCUGAA	UGCCAGGGUAGUAGUCUAAUUGGUAUAGCUGAAGGGCUGAUGGUAACGCAUAGCCCUUCAGCAAUACCAAUUAGACUACCACCCUGGCU	26.96	24.71	24.71	23.59	1.091	1.047
ace-miR8752	gra-miR8752	UGAUGGAGAUAGGUAUCUGCA	UGAUGGAGAUAGGUAUCUGCA	AAUGAUGGAGAUAGGUAUCUGCACUCCGCAAACAAAACCACAUGCCUUGCUAUUCCCAGUCAAAAAGGACCCAGCAACACUUGGCGGGUAGUUUGAUGGCGGGUGAUGGUGUUAGAGAUAUGUGGUUUUGUUUGCGGAGUGUAGAUUCCUAUCGCCAACAUA	27.16	26.54	26.54	19.75	1.023	1.343

**Table 3:** Statistical analysis of miRNA parameters

Parameters	Minimum	Maximum	Average	Standard deviation
Length (nt)	89	200	146	30.77
(A+U)%	39.5	73.5	57.12	9.32
(G+C)%	26.41	60.5	42.88	9.32
A%	21	32.07	26.75	3.44
U%	19.5	41.5	30.46	7.17
C%	9.43	33	20.57	6.05
G%	16.15	26.14	22.18	3.8
A/U	0.615	1.142	0.911	0.189
G/C	0.803	1.8	1.14	0.298
MFE	-142.3	-48.71	-76.79	28.81
AMFE	-75.28	-36.31	-52.8	15.17
MFEI	-1.8	-0.89	-1.25	0.312

**Table 4:** Putative targets of the identified miRNAs from Allium cepa

miRNA name	Target Accn.	Target aligned fragment (5'-3')	Target Description	Expectation	UPE	Inhibition
ace-miR162	LOC_Os03g0121800	CUGGAUGCAGAGGCUUAUUGA	Dicer like protein (DCL)	1	14.1	Cleavage
	LOC_ Os03g0121901	CUGGAUGCAGAGGCUUAUUGA	Dicer like protein (DCL)	1	17.1	Cleavage
	LOC_Os03g38740	CUGGAUGCAGAGGCUUAUUGA	Dicer like protein (DCL)	1	17.8	Cleavage
ace-miR168	LOC_Os02g58490	GUCCCGAGCUGCAUCAAGCUA	AGO1 protein	1	11.23	Cleavage
	LOC_ Os02g0831600	GUCCCGAGCUGCAUCAAGCAA	AGO1 protein	0.5	17.1	Cleavage
ace-miR172c	TC374166	UUGCAGCAUCAUCAGGAUUCC	AP2-like ethylene-responsive transcription factor	0.5	11.51	Cleavage
	BP855546	AAGCUGCAUCAUCAAGAUUCA	Uncharacterized protein	1.5	13.96	Cleavage
	TC367780	AUGCAGCAUCAUCAGGAUUCU	AP2 domain transcription factor-like	0.5	16.3	Cleavage
	TC397650	UUGUAGCAUCAUCAGGAUUCC	AP2-like ethylene-responsive transcription factor	1	17.8	Cleavage
	TC393465	CUGCAGCAUCAUCAGGAUUCU	Floral homeotic protein APETALA 2	0.5	17.4	Cleavage
	LOC_Os06g43220	CUGCAGCAUCAUCAGGAUUCC	Floral homeotic protein APETALA 2,	1	15.4	Cleavage
	LOC_Os04g55560.1	CUGCAGCAUCAUCACGAUUCC	AP2 domain containing protein	1	9.3	Cleavage
	LOC_Os04g55560.2	CUGCAGCAUCAUCACGAUUCC	AP2 domain containing protein, expressed	1	14.2	Cleavage
ace-miR172e	TC385694	UGUGAAUUUUGAUGAUGUUGU	Uncharacterized protein	1	19.3	Cleavage
	LOC_Os03g03510.1	UGUGAGCCUUGAUGAUGUUGA	CIPK-like protein 1	0.5	16.2	Cleavage
	LOC_Os03g03510.2	UGUGAGCCUUGAUGAUGUUGA	CIPK-like protein 1	0.5	9.3	Cleavage
	LOC_Os07g06590.2	UGGGGUUCUUGAUGAUGUUGC	ML domain protein	1.5	21.2	Cleavage
ace-miR398	LOC­_Os04g0501000	CGUGGCCGACCUGAGAACUCA	60S ribosomal protein	1	23.4	Cleavage
	LOC_Os11g0168100	CGUGGCCGACCUGAGAACUCA	60S ribosomal protein	1	19.3	Cleavage
ace-miR400	LOC_ Os09g0413300	GUUACUUAUAAUACUCUCAUA	Pentatricopeptide repeat-containing (PPR1) protein	0.5	19.7	Cleavage
	LOC_ Os09g0413301	GUGACUUAUAAUACUCUCAUA	Pentatricopeptide repeat-containing (PPR1) protein	0.5	19.1	Cleavage
	LOC_Os07g0239600	GUGACUUAUAAUACUCUCUUA	Uncharacterized protein	0.5	16.6	Cleavage
ace-miR414	CF451171	UGAAUAUGAUGAUGAUGAUGA	ATP dependent helicase	1	11.3	Cleavage
	CF452115	UCAAUAUGAUGAUGAUGAUGA	dynein-like Rea1 protein	1	15.6	Cleavage
	CF450746	UGUAUAUGAUGAUGAUGAUGA	Uncharacterized protein	1	11.3	Cleavage
ace-miR6219	LOC_Os05g18294.5	AUGCCCAUCUUUAGUCCCGGUUG	transporter-like protein	1	14.9	Cleavage
	LOC_Os03g55740.1	AUAACCCCCCUUUAGUCCCGGUUU	prolamin	1	13.7	Cleavage
	LOC_Os06g32290.1	UCGUAACCCCCCUUUAGUCCCGGUUU	Uncharacterized protein	0.5	14.9	Cleavage
	LOC_Os11g06630.1	UCGUAACCCCCCUUUAGUCCCGGUUU	ribosome inactivating protein 1	1	14.9	Cleavage
ace-miR1134	LOC_Os09g14670.1	CUCUUCUUCUUGUUGUUGUUGGCG	Phosphoenol pyruvate carboxylase 2	1.5	16.6	Cleavage
	LOC_Os01g12720.1	CUCGUUCUUUUUGUUGUUGUUGUUG	receptor protein kinase PERK1	1.5	22.6	Cleavage
	LOC_Os01g66030.2	CGCUUGUUCUUGUUGUUGUUGUUG	MADS-box transcription factor 2	1	17.6	Cleavage
	LOC_Os01g66030.1	CGCUUGUUCUUGUUGUUGUUGUUG	MADS-box transcription factor 2	1	11.9	Cleavage
	LOC_Os10g39770.1	AGCUUCUUGUUGUUGUUGUUGUUG	RING-H2 finger protein	1	15.6	Cleavage
ace-miR1223	CF447935	GCUAGCUGUAUCAUUCUUCAA	Uncharacterized protein	0.5	17.1	Cleavage
ace-miR7725	TC384565	UGGCAAUGCAUGUGGUUUUCU	Uncharacterized protein	2	18.6	Cleavage
ace-miR8570	CF446735	UCUUCAUGGACGACUUCCAACU	Uncharacterized protein	1.5	16.2	Cleavage
ace-miR8752	LOC_Os02g08364.1	GACAGAUGCCUAGCUCCAUCA	protein phosphatase	1	12.03	Cleavage

**Table 5:** Gene ontology (GO) based functional classification of the predicted miRNA targets

GO IDs	Description	E-value	No. of genes	Accn. ID for the target	miRNAs
BIOLOGICAL PROCESS					
GO:0008152	Metabolic process	4.51E-05	10	LOC_Os03g0121800, LOC_ Os03g0121901, LOC_Os03g38740, LOC­_Os04g0501000, LOC_Os11g0168100, CF451171, CF452115, TC385694, LOC_Os03g03510, LOC_Os03g03510	miR162, miR172, miR398, miR414
GO:0019748	Secondary metabolic process	3.43E-05	5	LOC­_Os04g0501000, LOC_Os11g0168100, CF447935, TC384565, LOC_Os02g08364.1	miR398, miR1223, miR7725, miR8752
GO:0032502	Developmental process	3.27E-04	4	LOC_Os09g14670.1, LOC_Os01g12720.1, LOC_Os01g66030.2, LOC_Os01g66030.1	miR1134
GO:0006355	Regulation of transcription	1.33E-03	5	TC374166, TC367780, TC397650, LOC_Os04g55560.1, LOC_Os04g55560.2	miR172
GO:0023052	Signaling	5.17E-05	5	LOC_Os02g08364.1, LOC­_Os04g0501000, LOC_Os11g0168100, LOC_Os03g03510.1, LOC_Os03g03510.2	miR8752, miR398, miR172
GO:0006950	Response to stress	0.00097	4	LOC_Os02g58490, LOC_ Os02g0831600, LOC_ Os09g0413300, LOC_ Os09g0413301	miR168, miR400
GO:0002376	Immune system process	0.00173	2	CF451171, CF452115	miR414,
GO:0006952	Defense response	0.0281	2	CF451171, CF452115	miR414
MOLECULAR FUNCTION					
GO:0003676	Nucleic acid binding	2.47E-05	6	LOC_Os02g58490, LOC_ Os02g0831600, LOC­_Os04g0501000, LOC_Os11g0168100, CF447935, TC384565	miR168, miR398, miR1223, miR7725
GO:0005515	Protein binding	1.17E-05	2	LOC_ Os09g0413300, LOC_ Os09g0413301	miR400
GO:0003824	Catalytic activity	4.53E-04	7	LOC_Os02g58490, LOC_ Os02g0831600, LOC­_Os04g0501000, LOC_Os11g0168100, TC374166, TC367780, LOC_Os04g55560.1, LOC_Os04g55560.2	miR168, miR172, miR398
GO:0030234	Enzyme regulator activity	3.86E-03	3	CF451171, CF452115, LOC_Os02g08364.1	miR414, miR8752
GO:0003700	Transcription factor activity	2.89E-03	7	TC374166, TC367780, TC397650, TC393465, LOC_Os06g43220, LOC_Os01g66030.2, LOC_Os01g66030.1	miR172, miR1134
CELLULAR COMPONENT					
GO:0044464	Cell part	4.82E-04	5	LOC_Os03g0121800, LOC_ Os03g0121901, LOC_Os03g38740, LOC­_Os04g0501000, LOC_Os11g0168100	miR162, miR398
GO:0044424	Intracellular part	2.73E-03	3	LOC_Os03g0121800, LOC_ Os03g0121901, LOC_Os03g38740	miR162
GO:0043226	Organelle	2.66E-04	3	LOC_Os10g39770.1, LOC_Os09g14670.1, LOC_Os01g12720.1	miR1134
GO:0043227	membrane bound organelle	0.00176	2	LOC_Os10g39770.1, LOC_Os09g14670.1	miR1134
